# Enzyme Reaction Annotation Using Cloud Techniques

**DOI:** 10.1155/2013/140237

**Published:** 2013-09-26

**Authors:** Chuan-Ching Huang, Chun-Yuan Lin, Cheng-Wen Chang, Chuan Yi Tang

**Affiliations:** ^1^Department of Computer Sciences, National Tsing Hua University, Hsinchu 300, Taiwan; ^2^Department of Computer Sciences and Information Engineering, Chang Gung University, Taoyuan 333, Taiwan; ^3^Department of Applied Chemistry, National Chiao Tung University, Hsinchu 300, Taiwan; ^4^Department of Computer Sciences and Information Engineering, Providence University, Taichung 433, Taiwan

## Abstract

An understanding of the activities of enzymes could help to elucidate the metabolic pathways of thousands of chemical reactions that are catalyzed by enzymes in living systems. Sophisticated applications such as drug design and metabolic reconstruction could be developed using accurate enzyme reaction annotation. Because accurate enzyme reaction annotation methods create potential for enhanced production capacity in these applications, they have received greater attention in the global market. We propose the enzyme reaction prediction (ERP) method as a novel tool to deduce enzyme reactions from domain architecture. We used several frequency relationships between architectures and reactions to enhance the annotation rates for single and multiple catalyzed reactions. The deluge of information which arose from high-throughput techniques in the postgenomic era has improved our understanding of biological data, although it presents obstacles in the data-processing stage. The high computational capacity provided by cloud computing has resulted in an exponential growth in the volume of incoming data. Cloud services also relieve the requirement for large-scale memory space required by this approach to analyze enzyme kinetic data. Our tool is designed as a single execution file; thus, it could be applied to any cloud platform in which multiple queries are supported.

## 1. Introduction

Enzymes are biochemical agents that efficiently catalyze the conversion of substrates into products in organisms. Enzymes are essential to the metabolic activity of living systems, and they share 3 features: catalytic power, specificity, and regulation [1]. Catalytic power is the ratio of the rate of an enzyme-catalyzed reaction to the rate of the uncatalyzed reaction. Enzyme-catalyzed reactions provide faster rates than traditional biochemical processes because enzymes reduce the energy required for biochemical reactions. Enzymes perform specific actions, and their selection should be specific to the desired reaction; thus, the use of enzymes can avoid competing reactions from producing side products. Consequently, enzyme applications are increasingly being employed in industrial applications. Enzyme activities can be optimized to provide metabolic reaction rates that are appropriate to cellular requirements.

The catalytic power and specificity of enzymes can enhance productivity in industrial applications. A recent study published by the BBC research group estimated that the global market for industrial enzymes was at $3.3 billion in 2010 and was expected to reach $4.4 billion by 2015 [2]. Enzymes involved in digestion, such as lipase, protease, and amylase, are classed as hydrolases. The Nomenclature Committee of the International Union of Biochemistry and Molecular Biology (NC-IUBMB) classified enzymes into 6 groups: oxidoreductases, transferases, hydrolases, lyases, isomerases, and ligases. According to the NC-IUBMB scheme and the Enzyme Commission's (EC) system, an enzyme reaction is assigned a 4-numerical-block number [3]. The method presented in our study can facilitate enzyme annotation, and is also valuable in followups to biochemical studies and applications, including metabolic process investigations and drug discovery. 

There are 3 main types of enzyme reaction annotations: sequence similarity, chemical structure comparison, and domain architecture fingerprint. Certain annotation methods, such as profils pour l'identification automatique du métabolisme (PRIAM) [4] and Catalytic Families (CatFam) [5], are based on protein sequences. These methods generate high-level profiles from sequences to represent and determine protein catalytic functions. The EnzymeDetector [6] annotation method uses sequence similarity analysis and a comprehensive enzyme database, BRaunschweig ENzyme DAtabase (BRENDA) [7], which is manually extracted from the literature. The Enzyme Function Inference by Combined Approach (EFICAz) [8] method adopts and combines various independent sequence-based methods. 

The second type of enzyme reaction annotation is based on chemical structure comparison because the conversion of a particular reactant into a product with a specific molecular structure in an uncatalyzed chemical reaction can often be achieved by enzyme catalysis in an organism. Problems are frequently encountered when an enzyme catalyzes several reactions and when the same reaction is catalyzed by different enzymes. Several reported computational methods exist for assigning EC numbers that use the physicochemical and topological properties of reactants, products, and bonds involved in the reaction [9–12]. 

Domain architecture fingerprint is the third type for enzyme reaction annotation. Substrates bind to an enzyme at its active site, where they undergo reaction. An enzyme reaction is intimately linked to the compact protein structure of a domain. As a general rule, enzymes of similar domain architectures catalyze similar reactions; this creates a difficult mapping problem from the architecture space into the reaction space. Various machine-learning methods have been applied to the mapping problem, including the association rule algorithm [13], the decision tree method [14], support vector machines [15], neural networks [16], and other classification schemes including domain teams [17], probabilistic rule-based models [18], and a weighted domain architecture comparison tool, the Feature Architecture Comparison Tool (FACT) [19].

The advent of genomics technologies, including next-generation sequencing and mass spectrometry-based flow cytometry [20, 21], creates an exponential growth in the volume of data. Cloud technologies provide large computing capacity, and this allows for the integration of distributed large-scale facilities for managing user requests and providing cost-efficient responses. Platform as a Service (PaaS) is provided by several companies, including Google, Microsoft, and Amazon. Microsoft's DryadLINQ execution engine and its application to the Alu clustering problem and an expressed sequencing tag (EST) assembling program in Apache Hadoop are extensions of the Google MapReduce platform [22]. Our proposed scheme requires large-scale computer memory for estimating and ranking each subset based on the domain architecture enumeration phase measurements. The results of queries when using this scheme are efficiently managed by the cloud's distributed architectures. Because adopting cloud technology enables annotation schemes to provide new architecture, the global enzyme market is expected to benefit from the increases in production capacity made available by the new architecture.

## 2. Materials and Methods

Proteins comprise polypeptide chains that form several compact, occasionally loosely connected, global units called structural domains. Regarding the protein structure, structural domains are considered fundamental units of protein function, folding, and evolution [23]. It is reasonable to consider a protein as one type of domain architecture consisting of a set of domains. The SUPERFAMILY structural domain database, integrated into the InterPro database (release 33.0), is adopted for constructing the domain architectures of proteins. For example, a Q5VT25 protein consists of the domain architecture with the SUPERFAMILY domains SSF50729, SSF56112, and SSF57889, such that the set {SSF50729, SSF56112, SSF57889} is considered to represent Q5VT25. Moreover, different proteins may share the same domain architecture of {SSF50729, SSF56112, SSF57889}, such as Q9BZL6 and E0W264. A particular reaction may be catalyzed by different enzymes, and an enzyme can often mediate more than one reaction. The resulting complex relationship between the set of domain architectures and the set of enzyme reactions remains a difficult problem, even after simplifying by considering a protein as one type of domain architecture. In this study, we identified proteins and recorded their corresponding domain architectures and enzyme reactions in our database.

### 2.1. Data Sets

From the viewpoint of protein function, enzymes are agents of metabolic function, which control the rate of biochemical activities in living organisms [1]. The first block of the EC number indicates to which of these 6 groups an enzyme belongs. The second and third blocks indicate subclass and sub-subclasses according to the enzyme's associations with the chemical features of the reactants and products of the reaction system. The final block is a sequential number. Enzymes are collected based on their corresponding EC numbers from the UniProt Knowledgebase (UniProtKB), such as Q5VT25 associated with EC 2.7.11.1, Q9BZL6 with 2.7.11.13, and E0W264 with 1.3.1.74 and 2.7.11.13.

UniProtKB [24] is a comprehensive protein sequence and annotation resource. It comprises UniProtKB/Swiss-Prot and UniProtKB/TrEMBL sections. The literature-based records in the Swiss-Prot section are manually annotated and analyzed computationally by curators. The TrEMBL section contains records that are annotated automatically, using qualitative computational analysis methods. Enzyme reactions described by either UniProtKB/Swiss-Prot or /TrEMBL are collected. The InterPro [25] database is an integrated resource of protein signatures in which protein domains held in different member databases are cross-referenced. We used the SUPERFAMILY member database [26] to investigate the relationship between domain architectures and enzyme reactions. All enzymes assigned EC numbers were collected from the Swiss-Prot and TrEMBL sections of UniProtKB (release 2011_07). We extracted the proteins that (1) had specific EC numbers and (2) were cross-referenced to SUPERFAMILY (version 1.73) in the InterPro database (release 33.0). Based on the integrated material we gathered from the UniProtKB and SUPERFAMILY databases, there are totally 1,664,839 proteins composed of 1,218 SUPERFAMILY domains and 3,306 related EC numbers.

**Figure 1:**
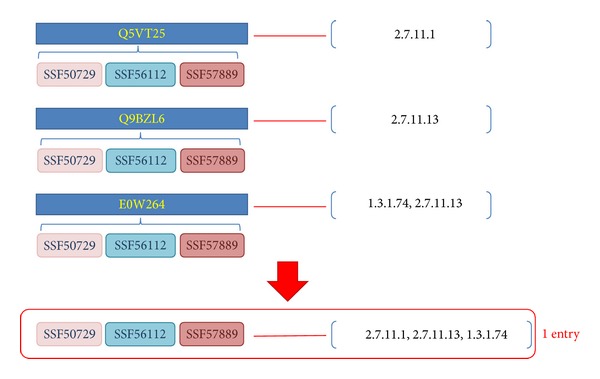
Illustration of an entry.

Relying on the rationale that structural domains are related to protein functions, we integrated enzymes sharing the same domain architecture as a single entry. For example, Q5VT25, Q9BZL6, and E0W264 share the {SSF50729, SSF56112, SSF57889} architecture with EC numbers 2.7.11.1, 2.7.11.13, and 1.3.1.74 and are considered a single entry (Figure 1). There are 5,203 entries collected in this study, and each entry consists of one type of domain architecture associated with several enzyme reactions.

Our proposed method accounts for the frequency of each potential type of domain architecture from a set, and a rank is assigned according to several criteria. After determining the domain architecture that has the greatest score, we obtain the corresponding enzyme reactions.

### 2.2. Methods

Because domains are fundamental structural units that can fold into a compact block, we considered the appearance of a domain in an enzyme and omitted the repetition of domains. As a result, the number of domain architectures is nearly 5 times the number of types of SUPERFAMILY domains but does not grow exponentially. This shows a tendency for one domain to accompany others to form one type of domain architecture for a protein. The ERP method is used to predict enzyme reactions from components of domain architectures. In the model-building process, there are 2 main phases: “domain architecture enumeration” and “enzyme reaction ranking.”

Before building the prediction model, we divided 5,203 entries into 2 sets, the training set and the testing set. The training set is used to establish the prediction model and the testing set is adopted for verification. The details of the model simulation are described in the 5-fold cross-validation section.

#### 2.2.1. The Enzyme Reaction Prediction Method

The first phase of model building is based on the rationale that one domain has a tendency to accompany others to form one type of domain architecture. We enumerated all possible subsets from domain architectures in the training set and estimated each subset according to 4 measurements: comprising existence, succinctness, consistency, and simplicity. The domain architecture candidate with the highest priority was thus obtained. In the second phase, we ranked enzyme reactions in a list according to their intensity values associated with one specific type of domain architecture.

**Figure 2:**
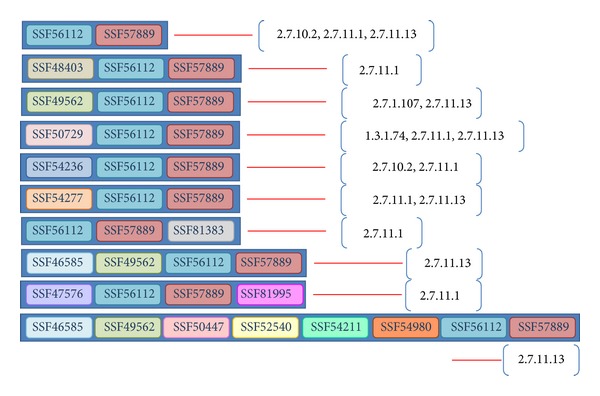
Entries containing domains SSF56112 and SSF57889.

**Figure 3:**
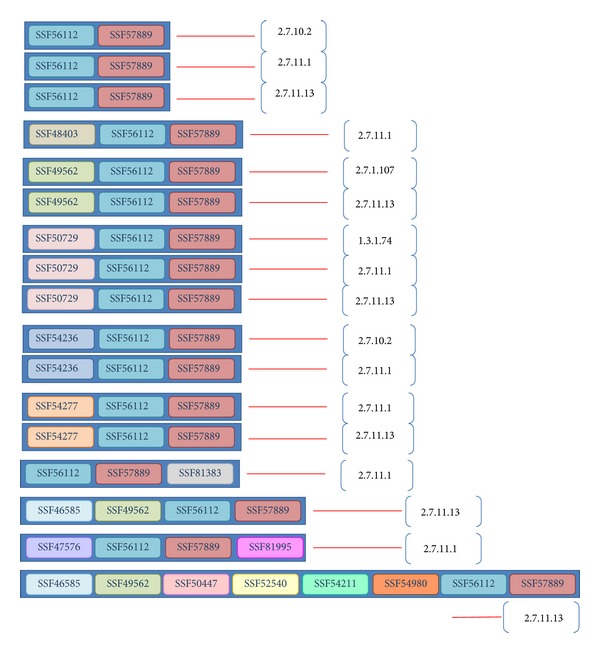
Separating entries into certain types of an architecture with one EC number.

*Domain Architecture Enumeration. *After inspecting the set of domain architectures, we learned that the number of types of adjacent domains was considerably lower than the numbers encountered when enumerating every possible combination. Thousands of architectures are enumerated exhaustively among all the possible subsets associated with the domain architectures of proteins. A possibility of tandem domains appearing with the expression of enzyme-catalyzed reactions also exists. Thus, we propose the following 4 measurements to sequentially estimate each domain's architecture during the domain architecture enumeration phase. For example, if the domain architecture {SSF50729, SSF56112, SSF57889} could not be found, 6 subarchitectures comprising {SSF50729, SSF56112}, {SSF50729, SSF57889}, {SSF56112, SSF57889}, {SSF50729}, {SSF56112}, and {SSF57889} are considered.

(*1) Existence of the Protein Consisting of a Given Domain Subset*. In the process of enumerating all possible subsets of domain architectures, many putative subsets may be produced. If one subset matches one type of domain architecture of an enzyme, it is reasonable that this domain subset contributes directly to its catalyzed reactions and is awarded higher priority than other subsets are.

(*2) Succinctness Measurement of the Domain Architecture of Enzymes. *One reaction can be catalyzed by various enzymes that can comprise a variety of domain architectures. Among them, each subset of one type of domain architecture could also include another type of an enzyme. The Succinctness_domain_arch_ equation (1) is designed to identify the most relevant architecture. Given an enumerated domain subset called domain_arch, we collected a set of entries, Entries_domain_arch_, that have domain architectures containing domains in domain_arch. The number of reactions associated with the entries which have domain architecture that exactly match domain_arch is denoted as |ECs_exact_|. The number of reactions associated with the entries that have architectures containing domains in domain_arch is denoted as |ECs_included_|. The Succinctness_domain_arch_ measurement is calculated as the ratio of |ECs_exact_| to |ECs_included_|. The type of domain subset with a greater Succinctness_domain_arch_ value is assigned higher priority among a set of architecture candidates for the query domain architecture. For example, a query architecture domain_arch consisting of domains SSF56112 and SSF57889 is involved in 10 entries involving 5 types of enzyme reactions, comprising 2.7.10.2, 2.7.11.1, 2.7.11.13, 2.7.1.107, and 1.3.1.74 (|ECs_included_| = 5) in Figure 2. The exactly matched architecture {SSF56112, SSF57889} is associated with 3 reactions, 2.7.10.2, 2.7.11.1, and 2.7.11.13, such that the Succinctness_{SSF56112, SSF57889}_ is estimated as 0.6. We assign priority to the candidate with the greatest succinctness value because the corresponding chemical reactions proceed without requiring auxiliary domains as follows:

(1) $$\begin{array}{c} \text{Succinctnes}{\text{s}}_{\text{domain}\_\text{arch}}=\frac{\left|\text{EC}{\text{s}}_{\text{exact}}\right|}{\left|\text{EC}{\text{s}}_{\text{included}}\right|}. \end{array}$$

(*3) Multiplicity of Enzyme Reactions from One Type of Domain Architecture*. An enzyme can catalyze different reactions; alternatively, different enzymes may share the same domain architecture. Considering a domain subset domain_arch, we collected all entries that have domain architectures containing domains of domain_arch. Among these entries, the number of involved reactions is defined similarly to the definition of |ECs_included_| in the previous paragraph, but we denoted it as *k* for simplicity. To clearly observe the expression of one specific reaction among various architectures, we separated an entry with multiple reactions into several entries with a single reaction, and the number of entries with a single reaction is counted as *N* (Figure 3). Furthermore, we also mark the number of entries associated with each reaction EC_*i*_ as *n*_*i*_ (*i* = 1, …, *k*), such that *N* = ∑_*i*=1_^*k*^ *n*_*i*_. The mean value $\bar{n}=N/k$ is calculated as the average number of entries, and the difference $\left(n_{i}-\bar{n}\right)$ is estimated for each reaction EC_*i*_. Because *k* and Entries_domain_arch_ are variables dependent on the set of domains in domain_arch, we provided Consistency_domain_arch_ (2), which summarizes the different terms and is normalized by *N* and weighted with (*n*_*i*_/*N*) for each reaction for comparison with other architecture candidates.

If the expression of each reaction is equal, then *n*_*i*_ approaches the mean value, such that the consistency value becomes smaller. As the consistency value approaches zero, it unambiguously indicates a strong relationship between enzyme-catalyzed reactions and the corresponding domain architecture:

(2) Consistencydomain_arch=n1N(n1−n¯N)+n2N(n2−n¯N)+⋯+nkN(nk−n¯N)=∑i=1kniN(ni−n¯N),

(3) n1=n2.7.10.2=2n2=n2.7.11.1=7N=∑i=1kni=17 (where  k=5)n3=n2.7.11.13=6⇒n4=n2.7.1.107=1n¯=Nk=175,n5=n1.3.1.74=1

(4) Consistency{SSF56112,SSF57889}  =n1N(n1−n¯N)+n2N(n2−n¯N)+n3N(n3−n¯N)   +n4N(n4−n¯N)+n5N(n5−n¯N)  =∑i=15niN(ni−n¯N)  =217(2−(17/5)17)+717(7−(17/5)17)   +617(6−(17/5)17)+117(1−(17/5)17)   +117(1−(17/5)17)  =0.114878892733564.

(*4) Simplicity of Domain Architecture*. In the case that no protein matching the query architecture domain_arch is found, the fewest number of domains in an architecture candidate is preferred.

The aforementioned 4 measurements for 6 subsets of the domain architecture {SSF50729, SSF561112, SSF57889} are listed in Table 1. Because each subset has the same domain architecture as another protein, the subset {SSF56112, SSF57889} with the highest succinctness value of 0.6 has the highest priority. 

**Figure 4:**
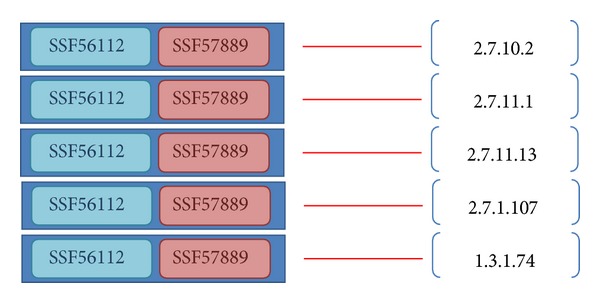
EC numbers connected with the architecture {SSF56112, SSF47889} by the ERP method.

*Enzyme Reaction Ranking*. After determining the domain architecture for a nonannotated enzyme, various related enzyme reactions can be retrieved from the universe data set (Figure 4). The Intensity_EC_*i*__ (5) is calculated based on the ratio of the mean value to the number of entries associated with EC_*i*_ to evaluate the strength of the relationship between the reaction EC_*i*_ and the determined domain architecture. An Intensity_EC_*i*__ value of less than 1 indicates that the expression of EC_*i*_ is greater than the mean value; thus, low values are preferred. The intensity values corresponding to reactions of the domain architecture {SSF56112, SSF57889} are calculated as follows:

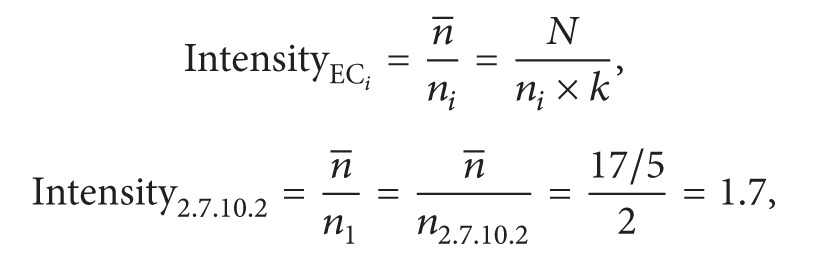
(5)

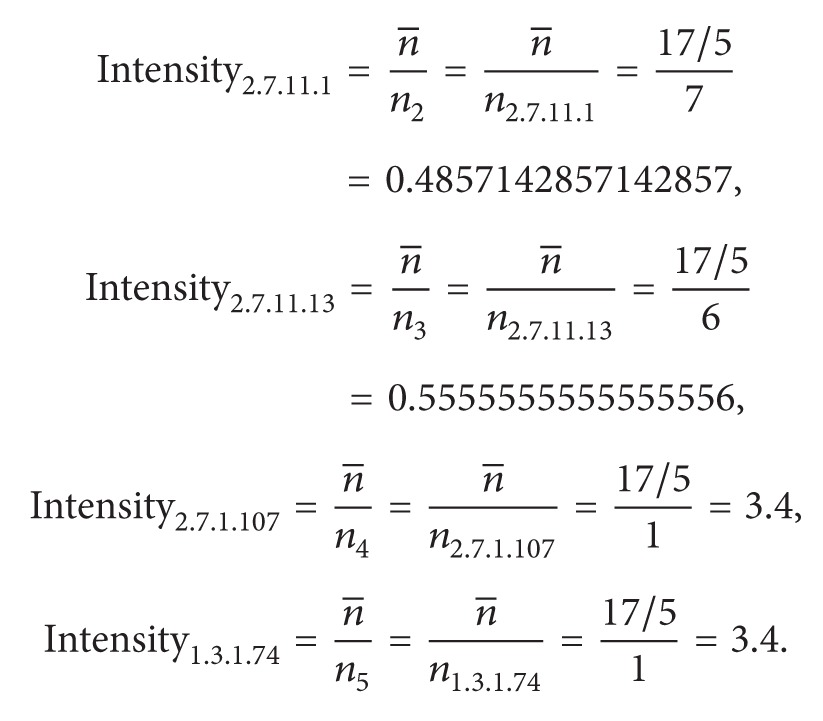
(6)


#### 2.2.2. The Association Rule Method

In the field of data mining, the association rule (AR) method is an established method for detecting the relationship between items, particularly for a large database, *T*. Given a large transaction set, if 2 sets, *X* and *Y*, are involved in a rule, *X* → *Y*, 2 constraints must be met: (1) the union of item sets *X* and *Y* must appear frequently in *T*, and (2) the relationship between item sets *X* and *Y* is close. A frequent set satisfies the condition that the number of transactions containing that set is higher than the support threshold. If set *Y* accompanies set *X* in various transactions, a close relationship exists between sets *X* and *Y*. The confidence value can be estimated as the ratio of the number of transactions containing both item sets (*X* and *Y*) to that containing item set *X* alone. If the confidence value of the item sets in a rule is higher than the given confidence threshold, it is placed into the rule set.

#### 2.2.3. Fivefold Cross-Validation

In a classification model, the parameters of the model are optimized to fit the training set as much as possible during the fitting process. An overfitting problem results when another independent validation data set (from the same population) is used to test the model and does not fit as well as the training set did. Cross-validation is a technique used to infer the goodness of fit of a model to a validation set. We used 5-fold cross-validation, in which the sample is randomly divided into 5 subsets: one subset is retained as the testing set, and the other subsets are assigned to the training set. The numbers of entries in each fold for the 4-numerical-block EC number set are shown in Table 2. One round of 5-fold cross-validation involves taking one part as the testing set and the remainder as the training sets, resulting in 20 total rounds of testing.

## 3. Results and Discussion

A chemical reaction may be catalyzed by more than one enzyme, and an enzyme may catalyze more than one reaction. By considering the relationship between enzymes and chemical reactions as a mapping problem, we create a many-to-many mapping problem. Although there are various methods available that approach this type of problem from different viewpoints, we present this intuitive method, which is based on the frequency of domain architecture and, in an enzyme, the associated catalyzed reactions. 

**Figure 5:**
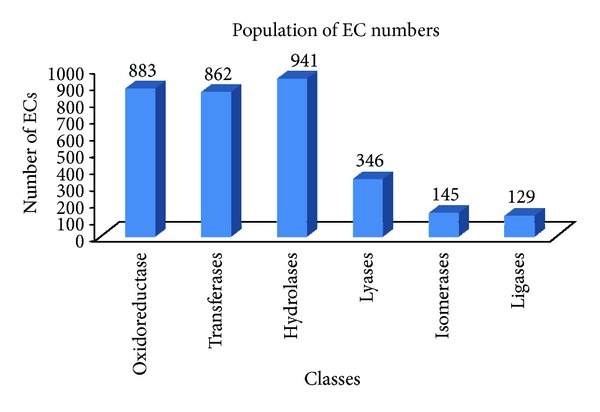
Population of EC numbers in the universe data set according to the six NC-IUBMB classes.

To examine the feasibility of our method, we compiled data from the UniProtKB and SUPERFAMILY domains of the InterPro database. A total of 1,664,839 proteins are associated with 1,218 SUPERFAMILY domains and 3,306 4-numerical-block EC numbers. The population of the 6 NC-IUBMB classes is shown in Figure 5. If one type of domain architecture was only associated with one enzyme reaction, then we collect these entries as a single-EC set. Entries associated with more than one enzyme-catalyzed reaction were assigned to a multiple-EC set. There were single-EC entries and multiple-EC entries in both the training set and the testing set. The ratio of the number of single-EC entries to the number of the multiple-EC entries in the testing set was approximately 6 : 4. Detailed information is shown in the “Testing set” column in Table 3.

To avoid the bias caused by the selection of the training data set, we used 20 runs of 5-fold cross-validation. From 5,203 entries, approximately 4,160 entries were used for model building, and the remaining 1,040 entries were used for verification. According to the complexity of classification problems, it is difficult to predict multiple reactions of entries from domain architectures. We separated 1,040 entries into 2 sets: 624 entries for the single-EC set and 416 entries for the multiple-EC set; hence, there are 2 main rows in Table 3. If an entry's domain architecture could be determined by a model, it indicated that the entry could be predicted by the model and it would be counted in the “Match” column. The “Hit” column records the number of entries that were predicted correctly. 

For comparison with our ERP method, we used the established Apriori algorithm [27] to mine for ARs implemented in a data mining package, Data-Mining-AssociationRules-0.10, of the Comprehensive Perl Archive Network (CPAN) [28]. The support and confidence threshold values used according to Chiu's settings [13] were 3 and 0.6, respectively. Table 3 shows that entries that were predicted using the AR method were considerably fewer than those predicted using the ERP method. To compare the 2 methods fairly, the same testing sets were used in the “AR” and “ERP1” rows, and entry sets that could be matched using the AR method were used as the testing set in row “ERP2.” When more entries were predicted using the ERP method (the “ERP1” column), it resulted in a lower prediction rate than when using the AR method (the “AR” column) in Table 4. However, the ERP method is slightly more effective when considering entries that could be predicted using the AR method (the “ERP2” column). 

The accuracy is provided by the ratio of the number of entries predicted correctly to the number of entry-matching rules of each method in Table 4. After 20 runs of 5-fold cross-validation, the mean accuracy values for 100 simulations were estimated. In a single-EC case, both the AR and ERP results reached 90%. However, estimation was less accurate for multiple-EC reactions. It is worth mentioning that both the ERP1 and ERP2 results are higher than the AR method in the multiple-EC set. 

**Figure 6:**
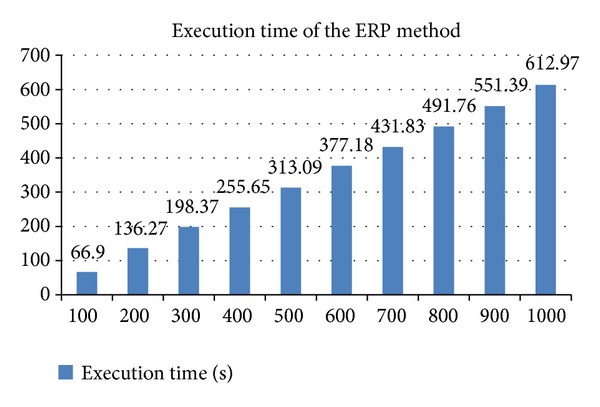
The average execution time of the ERP method for 100 simulations.

In the model-building phase, we implemented the AR method in a server equipped with 12 CPUs (4 cores, 3 packages) and 128 GB of memory, and the server used for the ERP method was equipped with 2 CPUs (2 cores, 1 package) and 8 GB of memory. The average model-building time was over 1 hour for the AR method and 15 minutes for the ERP method. The reasons may be that the AR method needed to produce frequent item sets and many redundant rules was generated. Furthermore, estimates of the prediction time for a batch of query domain architectures are shown in Figure 6. The vertical axis indicates the execution time in seconds, and the horizontal axis marks the number of entries in a batch query.

**Figure 7:**
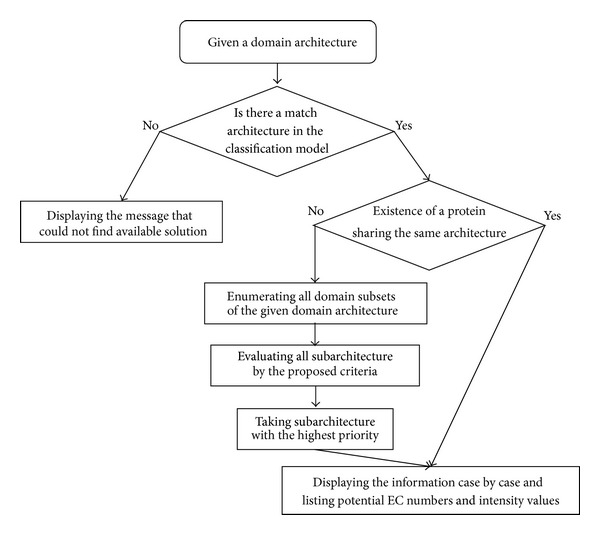
Workflow of querying a domain architecture in the ERP model.

A substantial demand exists for enzymes for industrial and medical applications in the global market; thus, enzyme function annotation is receiving considerable attention because it offers reductions in the cost of chemical processes. In this study, we proposed the ERP tool for annotating enzyme reactions based on the query domain architecture (Figure 7). After providing the domain architecture of a protein, the tool is used to determine whether available enzyme reactions exist; if not, an absence message is displayed. If enzyme reactions are available, the ERP tool is used to locate one type of the same domain architecture such that the corresponding enzyme reactions could be obtained with confidence. If the same architecture is not found, the next most promising subset is chosen from the given domain architecture, and its corresponding enzyme reactions are provided. If a similar domain architecture or a domain subset exists, proteins consisting of this architecture are displayed.

To implement the deduction of enzyme reactions from the domain architectures of enzymes, we designed a tool by using the Perl script language as follows. The set of domains in a protein must be listed before applying the ERP method. In the “Domain set” dialog, the domain set may be comma-or space-delimited. When the domain set is ready, pressing the “Predict” button starts processing according to the flowchart in Figure 7.

Two main situations in which analysis of the entered domain set could fail are described as follows.

**Figure 8:**
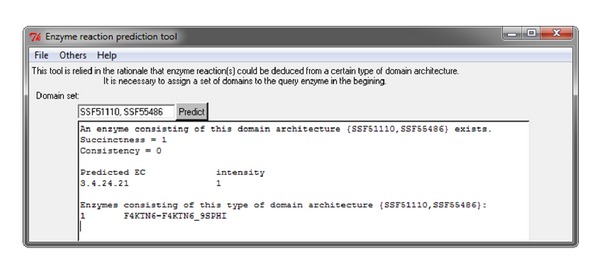
Message of the failure case for the domain architecture {SSF54211, SSF54236}.

1. If the ERP tool cannot deduce the corresponding enzyme reactions from the ERP integrated universe set, a failure message, such as the domain architecture failure notice {SSF54211, SSF54236} shown in Figure 8 is displayed in the results dialog, indicating that enzyme reactions associated with the query domain architecture could not be deduced from the universe data set.
2. In deducible cases, the existence of enzymes sharing the same architecture is considered. If the corresponding protein exists, succinctness and consistency values expressing the strength of the domain architecture are listed. If no enzyme sharing the same architecture is located, subsets of the domain architecture are evaluated, and the domain subset with the highest priority is selected.

**Figure 9:**
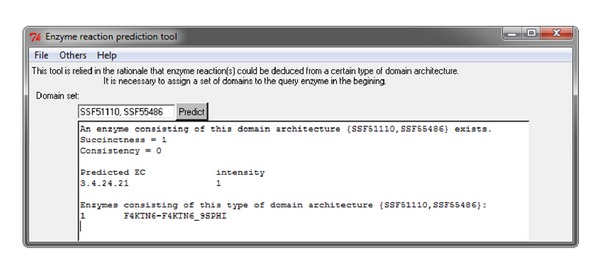
The case of existence of the same architecture protein for the domain architecture {SSF51110, SSF55486}.

In the event that the same architecture protein (Figure 9) is found, a confirmation message is displayed and the domain architecture (Figure 9, {SSF51110, SSF55486}) is identified. The succinctness value of 1 indicates that an enzyme with this type of domain architecture is capable of catalyzing the reaction denoted as 3.4.24.21 without any auxiliary domains. The consistency value of 0 indicates that a strong relationship between the domain architecture {SSF51110, SSF55486} and enzyme reaction 3.4.24.21 exists and that an association with other enzyme reactions does not exist. Because only one associated enzyme reaction exists, the strength measurement Intensity_3.4.24.21_ is calculated as 1. The protein consisting of the architecture {SSF51110, SSF55486} is shown in Figure 9 as accession number F4KTN6 and UniProt ID F4KTN6_9SPHI. 

**Figure 10:**
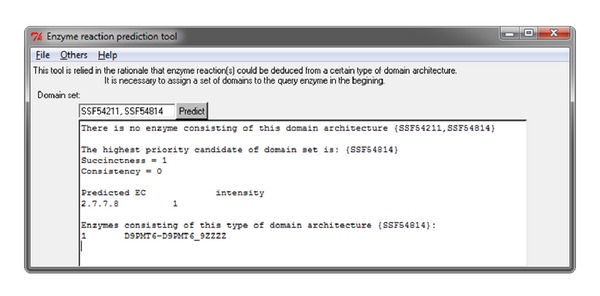
The report displayed in the case of an absence of any protein for the domain architecture {SSF54211, SSF54814}.

In the absence of a protein consisting of the same architecture (Figure 10), the subsets of domain architecture {SSF54211, SSF54814} are enumerated as {SSF54211} and {SSF54814}. After evaluating the 4 measurements used for enumerating the domain architecture, the candidate with the highest priority {SSF54814} is obtained. Similarly, an enzyme with this architecture is capable of catalyzing the reaction 2.7.7.8 independently and with succinctness value of 1. The relationship between the domain set {SSF54814} and enzyme reaction 2.7.7.8 is strong according to the consistency value of 0. Only one reaction, 2.7.7.8, is related to {SSF54814}; thus, Intensity_2.7.7.8_ is calculated as 1. The protein with the accession number D9PMT6 and UniProt ID D9PMT6_9ZZZZ consisting of this type of domain architecture {SSF54814} is listed in Figure 10.

## 4. Conclusion

In this study, we investigated the intimate relationship between domain architecture and enzyme-catalyzed reactions by applying various criteria to the compiled universe data set of domains and EC numbers. The advent of high-throughput techniques has produced numerous gene sequences, and annotating each enzyme reaction based on experimental results is difficult. However, we can consider domains as segments of sequences that fold into compact structural units; thus, we can model protein sequences and structures as these folded domains. We can identify and retrieve domains by integrating established sequence alignment tools with the proposed ERP tool.

## Figures and Tables

**Table 1:** Four measurement values for the six subsets of the domain architecture {SSF50729, SSF56112, SSF57889}.

Domain architecture	Existence	Succinctness	Consistency	Number (domains)
{SSF50729, SSF56112}	1	0.45	0.0688271604938272	2
{SSF50729, SSF57889}	1	0.5	0.058641975308642	2
{**SSF56112, SSF57889**}	**1**	**0.6**	**0.114878892733564**	**2**
{SSF50729}	1	0.191489361702128	0.0559722260571086	1
{SSF56112}	1	0.595744680851064	0.0765587606003442	1
{SSF57889}	1	0.4	0.11141975308642	1

**Table 2:** The number of entries in each fold.

Data set	Fold_1	Fold_2	Fold_3	Fold_4	Fold_5	Total
4-numerical-block EC number set	1,041	1,041	1,041	1,040	1,040	5,203

**Table 3:** The average number of entries for 100 simulations.

Data set	Method	Hit	Match	Testing set
Single EC	AR	25.36 ± 4.49	27.92 ± 4.43	624.60 ± 15.15
	ERP1	298.95 ± 13.12	592.53 ± 14.93	624.60 ± 15.15
	ERP2	25.82 ± 4.47	27.92 ± 4.43	27.92 ± 4.43

Multiple ECs	AR	3.76 ± 1.81	44.44 ± 5.02	416.00 ± 15.24
	ERP1	137.35 ± 11.72	378.61 ± 14.87	416.00 ± 15.24
	ERP2	18.47 ± 3.37	44.44 ± 5.02	44.44 ± 5.02

**Table 4:** Accuracy of the AR and ERP models.

	AR	ERP1	ERP2
Single EC	90.72% ± 5.71%	50.45% ± 1.85%	92.39% ± 5.22%
Multiple ECs	8.36% ± 3.80%	36.28% ± 2.74%	41.66% ± 6.62%
